# TransTCNet: Transformer-Based Temporal-Contextual Network for Low-Latency Typing Interfaces on Edge Devices

**DOI:** 10.3390/biomimetics11050337

**Published:** 2026-05-12

**Authors:** Asif Ullah, Zhendong Song, Waqar Riaz, Yizhi Shao, Xiaozhi Qi

**Affiliations:** 1 Shenzhen Institutes of Advanced Technology, Chinese Academy of Sciences, Shenzhen 518055, China; asifkh@szpu.edu.cn; 2 Institute of Ultrasonic Technology, Shenzhen Polytechnic University, Shenzhen 518055, China; yzshao@szpu.edu.cn; 3 Department of Computer Science and Engineering, School of Engineering, Nanfang College, Guangzhou 510970, China; riazwaqar@nfu.edu.cn

**Keywords:** surface electromyography (sEMG), typing recognition, deep learning temporal-contextual modeling, human–computer interaction, real-time neural interfaces

## Abstract

A distinct typing interface using surface electromyography (sEMG) can facilitate silent, hands-free typing by interpreting muscle activity in relation to specific keystrokes. Character-level recognition poses greater challenges than coarse gesture recognition because it is sensitive to subtle temporal variations and overlapping muscle dynamics. Temporal features are essential for typing recognition because keypresses may differ in duration, force, and accompanying hand movements across users. This paper proposes TransTCNet, a two-stage deep neural network architecture with a causal convolutional layer for learning local features and a transformer-based component for learning long-range temporal interactions. We evaluated our network on a publicly available 26-class typing sEMG dataset acquired from 19 individuals. The model achieved a validation accuracy of 96.53%, exceeding the baseline models. Our study revealed generalization among participants, and the AUC values were also high (>0.994) across all classes. The model was highly reliable and exhibited high prediction confidence (>0.9), enabling us to achieve a high training accuracy (97.86%) for real-time filtering decisions. TransTCNet could be suitable for wearable and edge devices due to its efficient architecture and low inference cost. The model’s ability to consistently decode fine-grained neuromuscular signals across users makes it well-suited for real-time applications such as adaptive user interfaces, virtual and augmented reality, prosthetic control, and communication systems.

## 1. Introduction

Effective interaction with complex computing systems requires high-throughput, intuitive channels to capture user intent. Even though conventional peripherals like keyboards, mice, and touchscreens are universal and well-established, new ideas in virtual, augmented, and mixed reality require more seamless, immersive interfaces that minimize the need for physical peripherals [1,2]. It can also be difficult to connect and communicate with physically disabled people using a standard gadget. Neurotechnology enables the direct decoding of user intent from neurological and neuromuscular cues, offering a promising approach to address these problems [3].

One of the many modalities of the sensory system is surface electromyography (sEMG). It is a non-invasive method for accurately measuring the electrical activity produced by skeletal muscles. sEMG was originally intended to control prosthetics [4] and has been used as a variety of sensing tools. More applications are being implemented for gesture recognition [5,6,7], facial expression analysis [8,9,10,11], handwriting replication [12,13], and, most recently, character-level typing recognition [14]. Such developments may have significant effects on human–computer interaction (HCI) when applied to problems where conventional input mechanisms do not work or cause interference.

One engaging application of sEMG is typing activities. The resulting sEMG patterns of every single keystroke during typing are frequently complex and delicate due to the highly fine motor activity. It is not simple to group and identify patterns when individual letter keys (A–Z) are treated as separate categories [14]. Addressing this issue could support the development of silent communication systems and sEMG-controlled virtual keyboards. Both technologies would be applicable across a broad spectrum of assistive technologies, immersive environments, and wearable devices.

Another field that has benefited from sEMG-based activity recognition systems is healthcare. Such systems can assist with physical therapy, track motor function, and enable control of myoelectric prostheses by clarifying muscle function. Machine learning is making these systems dynamic signal processors rather than mere signal processors [15,16,17]. Deep learning has revolutionized the field, eliminating the need to manually design features, and end-to-end spatiotemporal patterns can be learned from raw sensor data [18,19,20,21,22,23]. Deep learning models can also be effectively deployed in real-world systems, as they can adapt to user, sensor malfunctions, and environmental changes through continuous optimization. Recent healthcare-oriented studies have also demonstrated that compact transformer-based architectures combined with lightweight convolutional backbones can support real-time recognition tasks, further motivating the development of efficient attention-based designs for practical biomedical interfaces [24].

Several machine learning and deep learning techniques have been used to classify human activities from sEMG signals [25]. Al-Qaness et al. proposed Multi-ResNet, a multi-resolution residual network that employs attention mechanisms alongside residual neural networks to automate feature extraction from sensor data. The performance of this strategy is considerably better than that of the traditional HAR benchmark [26]. Similarly, Hnoohom et al. proposed an Att-BiLSTM network consisting of a convolutional and recurrent network with attention mechanisms that enable the recognition of spatial and temporal dependencies [27]. Choudhury et al. applied ensemble and shallow learning models to smartphone-collected data to achieve competitive accuracy levels [28,29]. These studies emphasize the growing diversity and sophistication of deep learning models used to simulate complex biosignals [30].

Nonetheless, most extant research remains constrained by fundamental boundaries. Primarily, sEMG signals are sensitive to sensor placement, muscle fatigue, physiological variations among individuals, and generalization across sessions, which is a recurring issue [31,32]. Second, for fine-grained classification tasks such as alphabet-level typing recognition, several models appear to be over-tuned to simple or static gestures [33,34]. Third, deep networks are prone to overfitting, especially when using small datasets [35]. Lastly, attempts to interpret decision boundaries and represent cognitive structure in a physiologically meaningful way have been minimal, thereby constraining interpretability [36].

This study aims to address gaps in current classification techniques for EMG signals recorded during keyboard use by leveraging more fine-grained feature sets. There are publicly available bilateral forearm EMG recordings from 19 subjects performing 26 distinct keyboard presses across two sessions [14]. The recordings include 16 channels of EMG data along with synchronized records of keyboard presses, thereby enabling the evaluation of different strategies across subjects and sessions. The objective of this study is to create a highly accurate/reliable classification model of keyboard presses with the intent of allowing for the creation of typing systems that interface with the sEMG derived from the movement of the user’s forearm.

Unlike coarse gesture recognition, character-level typing faces three distinct challenges: temporal variability in keystroke duration and force, muscle signal fusion among biomechanically similar keys (e.g., E–D, J–N), and sensitivity to electrode shifts across sessions.

**Figure 1:**
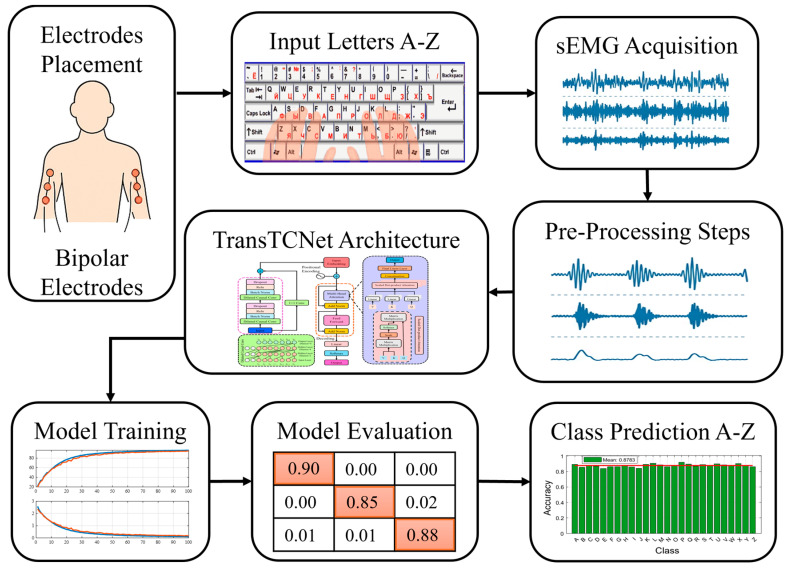
End-to-end pipeline for sEMG-based character-level typing recognition using a temporal-contextual deep learning framework.

Therefore, we present TransTCNet, a transformer-based model that generates temporal-contextual neural networks that learn short-term (temporal) and long-range sequential relationships from all EMG data generated during typing. By using attention-based encoding and dilated causal convolution, this model leverages long-range context across all input signals while quickly identifying localized patterns. The method, as described in detail in Figure 1, shows that our model was built specifically to classify fine-grained, character-level signals from the surface electromyography (sEMG) system (as opposed to some previous studies). It achieves optimal performance by recording 16 channels of bilateral forearm muscle activity with low latency and high sampling rate. We have demonstrated that this is the first model to utilize temporal attention and convolutional layers specifically for typing interfaces.

Transformers are particularly suited for this task. Unlike CNNs with limited receptive fields or RNNs with sequential processing and vanishing gradients, multi-head self-attention captures long-range temporal dependencies in parallel, enabling content-aware weighting across the entire 400-timepoint window while remaining compatible with low-latency implementation on resource-constrained devices.

Across 26 typing classes, our method outperformed alternative baseline models (e.g., 1D-CNN, CNN-SE, SE-TCN), achieving a maximum validation accuracy of 96.53%. Our findings indicate that temporal contextual models, such as those developed by the authors, have significant potential to enable users with disabilities to type adaptively using hands-free, wearable, or immersive devices.

## 2. Materials and Methods

### 2.1. Dataset Description

#### 2.1.1. Keyboard Typing sEMG Dataset

A publicly available surface electromyography dataset for character-level keyboard typing identification was used in this study [14]. The dataset description in this subsection, including the participant protocol, electrode configuration, sampling frequency, typing task, and acquisition-stage filtering, is based on the original dataset reported in the reference [14]. Fine-grained neuromuscular data captured during key presses enables future research on silent text entry, adaptive interfaces, and bioelectric control systems.

Participants performed a structured typing task using a standard QWERTY keyboard, with a unique class for each letter of the alphabet (A–Z), creating a 26-class classification problem. The study used a scheme to provide finger-to-key mapping, ensuring that participants maintained a stable typing posture throughout the typing trial.

The bilateral sEMGs (two forearms) were captured at 2000 Hz and bandpass filtered from 10 to 500 Hz using a 4th-order Butterworth filter during the original dataset acquisition stage to reduce motion artifact and high-frequency noise while preserving the frequency content of interest for motor control. Sixteen bipolar electrodes (8 on each arm) were used to collect the raw signals.

For every letter, the five presses of the spacebar before the trial served as an alignment mechanism so that each test trial had previously pressed the spacebar. All letter key presses were also recorded with a metronome set to 75 BPM, ensuring temporal consistency across subjects and repetitions. This pacing allowed participants to reduce mental workload and muscle fatigue, thereby improving the correspondence between sEMG spikes and keystrokes. It was an essential feature for constructing supervised models from data. However, the fixed timing used in the study will not account for inherent individual typing rhythms or fatigue from prolonged typing. Both factors significantly influence sEMG characteristics during normal daily tasks. Future studies could adopt free-typing paradigms and fatigue-related data to further improve ecological validity and generalizability.

The proposed methodology was completed for two repetitions of each letter in each subject testing session. Consequently, each letter had 20 repetitions per subject, resulting from completing the task in two separate sessions on two separate days, and remained for analysis. It is important to note that all keypress trials and related subject-key activities, i.e., background and idle sEMG, were temporally segmented. Consequently, only completed active-character-class (A–Z) data were available for analyses and could not include any “rest” class identification. Future work will require continuous sEMG acquisition to detect both the active class and the rest state, enhancing the capability for real-time applications.

#### 2.1.2. Participants

The dataset consists of 19 healthy individuals (5 men, 14 women) with a mean age of 31 ± 7 years. Ethical approval for the study protocol was granted by the University Health Network Research Ethics Board (Protocol #21-6137). Each subject participated in two recording sessions (T1 and T2), during which all alphabetic characters were recorded in trials. The experimental process enables the robust validity of session-based scenarios.

#### 2.1.3. Data Structure

The data were structured to facilitate model training and cross-session assessments. Raw sEMG signals were first recorded in proprietary electrophysiological formats and synchronized with the keystroke events, along with respective timestamp logs. These synchronized signals were then preprocessed and converted to standard NumPy arrays to enable effective processing in the subsequent machine learning pipeline.

The dataset was divided by subject and further subdivided into session-specific directories for structured access. Archiving pre-segmented 0.2 s signal windows that were temporally aligned to each keypress was executed so that they could be used in a window-based recognition. In addition, session-wise and user-wise feature sets were disclosed to facilitate generalization analysis, including intra-subject and inter-session generalization.

### 2.2. Data Preprocessing

**Figure 2:**
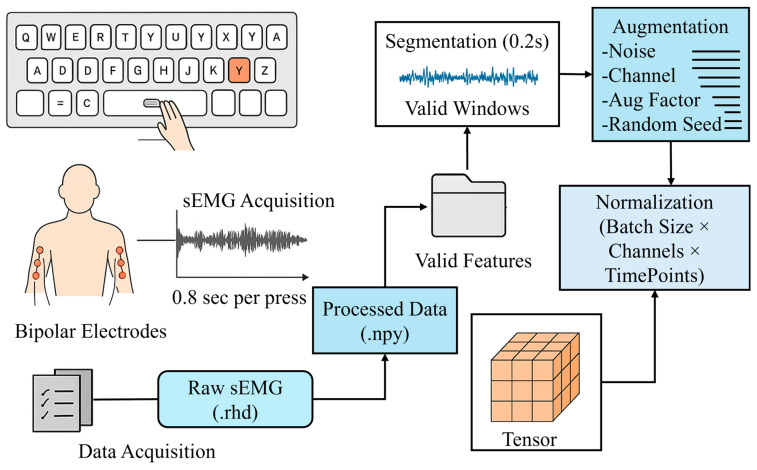
Overview of the sEMG preprocessing pipeline: from raw signal acquisition during alphabetic typing to segmentation, augmentation, normalization, and tensor formatting for model input.

The procedures described in this section represent the data preparation and refinement steps performed in the present study after obtaining the publicly available dataset from the reference [14]. The overall data-preprocessing process (as shown in Figure 2) comprises the basic steps for processing raw sEMG data into normalized tensors, which can then be used as input to the model. Before the classification dataset is prepared for model training, there are five main phases of pre-training data preparation: data curation, signal segmentation, signal augmentation, signal normalization, and signal reshaping.

#### 2.2.1. Valid Session and Window Selection

The first phase of data preparation involved determining and preserving whole, valid sessions (in which all 26 alphabetic classes were recorded correctly) and deleting trials that contained synchronization errors (or had no label), thus preserving an intact dataset. The signal windows for sEMG data were set to 0.20 s and aligned with keypress onsets. The segmentation method used ensured a high degree of temporal proximity between neuromuscular activity and the corresponding keypress gestures. Thus, classification performance will benefit from this segmentation method. The selection of a 0.20 s window length was ultimately supported by an analysis of classification performance that balanced gesture classification accuracy and response time. Classification accuracy increased slightly with window length; however, beyond 0.20 s, accuracy decreased. Also, because real-time systems require response window lengths of less than 0.30 s to support typical typing speeds (180+ keystrokes per minute), the use of a 0.20 s window length provides a compromise between providing high fidelity recognition and a low latency inference, all of which are critical for applications in the neural interface domain.

#### 2.2.2. Data Augmentation

To improve model generalization and preserve the physiological significance of the surface EMG samples, all original sample windows were augmented by a factor of 3. Each original window was retained, and two synthetic copies were generated. Following the original 10–500 Hz acquisition-stage filtering described in Section 2.1.1, an additional conservative 50–450 Hz bandpass filtering step was applied to the segmented windows during augmentation/preprocessing. Small-amplitude transient noise was then simulated by adding zero-mean controlled Gaussian noise with σ = 0.01 to each sample. Additionally, 1 of the 8 channels was set to 0 for each original/synthetic sample to simulate a temporary sensor dropout or electrode disconnection. These distortions were deliberately kept small to prevent the integration of spectral artifacts that might alter underlying neuromuscular activity. The random seed was fixed to ensure reproducibility of training operations. This conservative augmentation approach enhances stability against small acquisition artifacts (e.g., motion or impedance changes) and is physiologically realistic. Nevertheless, the protocol does not currently model spatial perturbations, including electrode shift, which is a significant source of performance loss in practice and should be validated using specific strategies in further investigation.

#### 2.2.3. Normalization and Tensor Formation

To ensure stability throughout the training process and a homogeneous contribution from all features, each window was normalized per channel to have zero mean and unit variance. The resulting normalized signals were then converted into a 3D array of size (batch size × channels × time points), thereby making them compatible with the deep learning architecture. High-resolution sEMG recognition depends on efficient learning of both local temporal variations and long-range sequence patterns that support tensor representation.

### 2.3. Neural Network Architecture

#### 2.3.1. TransTCNet Pipeline

**Figure 3:**
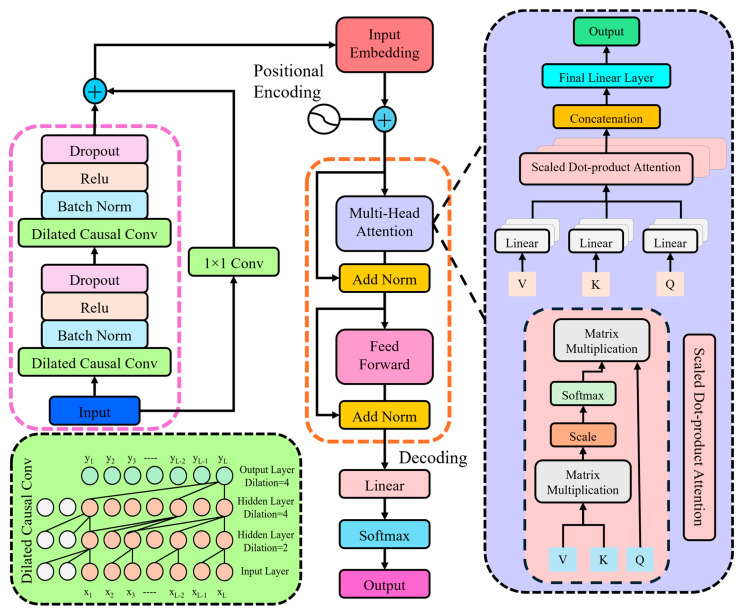
Architectural overview of the proposed TransTCNet framework, highlighting temporal feature extraction via dilated causal convolutions and contextual encoding using multi-head self-attention.

TransTCNet is a temporal-contextual deep learning network designed to efficiently and accurately detect fine-grained electromyographic (sEMG) signals during keyboard use. This technique examines multi-channel time-varying signals, primarily recording short-time variations and thereby storing a detailed sequence structure. As seen in Figure 3, the model encompasses two major steps:

##### Local Temporal Feature Encoding

The first part of TransTCNet focuses on extracting localized temporal features from raw sEMG signals using dilated causal convolutions. This approach ensures that the model assesses signal sequences in the inherent temporal sequence whilst allowing the receptive field to deepen without augmenting network depth.

The input to the network is a 3D tensor X ∈ R_B_ × C × T, where B is the batch size, C is the Number of EMG channels (e.g., 16), and T is the number of time samples per segment (e.g., 400). The data may be normalized and reshaped for 1D convolutional operations.

Short-range dependencies are extracted with the help of dilated causal convolutions. This operation ensures that the output at any given time depends only on the current and past inputs. Mathematically, the dilated convolution is represented as:

(1) $$y(t)=\sum_{i=0}^{k-1}w(i)\cdot x(t-d\cdot i)$$

where k is the kernel size, d is the dilation factor, and w(i) are learnable weights.

Each temporal block incorporates batch normalization to stabilize activations, ReLU activation for non-linearity, and dropout for regularization, along with residual connections to preserve gradient flow. When the input and output dimensions differ, a 1×1convolution is used to align them, which can be illustrated as

(2) $$X_{res}=Conv1\times 1(X)$$

This alignment is necessary because dilated causal convolutions with varying dilation factors can alter the effective receptive field and, in some cases, lead to inconsistencies in input and output channel dimensions. The 1 × 1 convolution serves as a learnable linear projection that preserves temporal resolution while matching the number of feature channels, enabling residual addition across layers. This operation ensures both shape compatibility and stable gradient propagation within the temporal block.

The output of the temporal block is denoted as:

(3) $$Z_{temp}\in R^{B\times C^{\prime }\times T}$$

where C′ is the number of output channels (e.g., 64), and T may be reduced due to padding and dilation control. This feature map is then passed to global contextual sequence modeling.

##### Global Contextual Sequence Modeling

After capturing local dependencies, the model’s next stage focuses on capturing long-range contextual information across the entire temporal sequence, which is critical for recognizing typing patterns distributed over time. First, the output tensor from the previous stage is reshaped to match the expected input format for the attention mechanism. The tensor, denoted as X_in_ ∈ R_B_ × C′ × T, where B is the batch size, C′ represents the number of output channels, and T denotes sequence length (time steps), is permuted to X_in_ ∈ R_B_ × T × C′, where each time step represents a feature vector t.

Next, a linear projection maps the feature vectors to a unified model dimension, dmodel, a hyperparameter that defines the model’s feature space (e.g., 128). This projection is mathematically expressed as:

(4) $$E=X_{proj}=X_{in}\cdot W_{proj}+b_{proj}\in R^{B\times T\times d_{model}}$$

where X_proj_ is the projection matrix, and b_proj_ is the bias term. This operation results in the projected tensor E ∈ R_B_ × T × d_model_, which is now in a form suitable for further attention-based processing.

Since attention mechanisms are permutation-invariant, meaning they do not consider the temporal order of the sequence, positional encoding is added to inject information about the relative or absolute positions of the time steps in the sequence. The positional encoding P is either learnable or sinusoidal, and it is added to the projected tensor E as follows:

(5) $$E_{pos}=E+P$$

where P ∈ R_1_ × T × d_model_ represents the positional encoding matrix, which allows the model to account for the temporal order of the sequence.

Afterward, a multi-head self-attention (MHA) layer is applied to enable the model to attend to multiple positions in the input sequence simultaneously, each step being represented by a different “head (h).” For each attention head, the mechanism computes a weighted sum of the input sequence, with weights derived from the query (Q), key (K), and value (V). Mathematically, the attention mechanism for a single head is defined as:

(6) $$Attention(Q,K,V)=Softmax(\frac{QK^{⊤}}{\sqrt{d_{k}}})V$$

where Q, K, V are linear projections of the input Epos and dk is the dimensionality of keys (usually d_k_ = d_model_/h). The result of this computation is a weighted sum of the values, which is the output of one attention head. This operation captures dependencies between positions (time steps) in the sequence by computing attention scores based on the similarity between queries and keys.

For multiple heads (denoted by h), the results from each head are concatenated, and a final linear projection is applied to get the final multi-head output. The operation is mathematically expressed as:

(7) $$MultiHead(X)=Concat(head_{1},\ldots ,head_{h})\cdot W^{O}$$

where each head_i_ is the output from a single attention head, and W^O^ is a learned weight matrix that projects the concatenated result back into the desired output dimension. This operation enables the model to capture temporal dependencies by assigning attention weights based on the similarity between time steps. Each head focuses on different aspects of the sequence, and their concatenation allows integration of diverse temporal patterns into a unified representation.

Next, layer normalization is applied to stabilize the learning process. It is achieved by normalizing the MHA layer’s output, adding it back to the input, and using a residual connection to preserve gradient flow during backpropagation. Mathematically,

(8) $$Z_{1}=LayerNorm(E_{pos}+MultiHead(E_{pos}))$$

Following this, a position-wise feedforward network (FNN) is applied independently at each time step in the sequence. The FFN consists of two linear layers with a ReLU activation between them. The mathematical operation is:

(9) $$FFN(z)=max(0,z\cdot W_{1}+b_{1})\cdot W_{2}+b_{2}$$

where W_1_, W_2_, and b_1_, b_2_ are the learned weight matrices and bias terms for the two linear layers. The ReLU activation introduces non-linearity to the network, enabling it to learn complex representations.

After the FFN layer, layer normalization is again applied to the output of the FFN and added to the input (residual connection), ensuring stability during training:

(10) $$Z_{2}=LayerNorm\left(Z_{1}+FFN\left(Z_{1}\right)\right)$$

After repeating this process for N layers (e.g., N = 4), we obtain a final tensor designated as,

(11) $$Z_{final}\in R^{B\times T\times d_{model}}$$

To convert this sequence into a fixed-size representation, temporal average pooling is applied to average the embeddings across all time steps, effectively summarizing the entire sequence into a single vector:

(12) $$F_{agg}=\frac{1}{T}\sum_{t=1}^{T}Z_{final}[:,t,:]$$

Finally, the aggregated features are passed through a linear classifier to obtain the output logits for each of the 26 alphabet classes. The output is computed as:

(13) $$y_{logits}=F_{agg}\cdot W_{cls}+b_{cls}\in R^{B\times 26}$$

where W_cls_ and b_cls_ are the weight matrix and bias term for the final linear layer. The result is a 26-dimensional vector of logits, which is subsequently passed through a softmax function to obtain the final probability distribution over the classes.

This two-stage design—combining local temporal feature extraction with long-range contextual modeling—enables TransTCNet to capture the intricate muscle activation sequences required for fine-grained sEMG-based character-level typing tasks. The model is designed to generalize well across different subjects and sessions, making it suitable for real-world applications such as muscle-driven text entry and assistive technologies.

Algorithm 1: TransTCNet summarizes the overall learning pipeline and describes the processes used during training and inference with the proposed model. The process begins with local temporal pattern extraction via dilated causal convolutions, followed by global context encoding via attention mechanisms. Training is based on the cross-entropy loss function and is optimized with the Adam optimizer. During the evaluation phase of the proposed model, accuracy and other performance metrics are calculated from the predicted probabilities.
**Algorithm 1:** TransTCNet Training and InferenceInput: Preprocessed sEMG windows X, class labels y, learning rate η, number of epochs N, batch size B Output: Trained model parameters θ*, evaluation metrics 1. Initialize TransTCNet parameters θ 2. Initialize optimizer (Adam) and loss function (Cross-Entropy) 3. for epoch ← 1 to N, do 4.      for each minibatch (X_b_, y_b_) ∈ D_train_, do 5.              Z_t_ ← DilatedCausalConv1D (X_b_) 6.              Z_c_ ← Self Attention (Z_t_) 7.              $\hat{y}$ ← Softmax (Classifier (Z_c_)) 8.              ℒ ← Cross Entropy ($\hat{y}$, y_b_) 9.                 θ ← θ − η ∇ θ ℒ end 10.      Model Evaluation (accuracy, F1-score, etc.) end Return: Final trained model θ*, performance metrics

## 3. Experimental Setup

### 3.1. Training and Validation Split

The data was stratified so that 80% was used for training and 20% for validation, ensuring that both subsets had the same class distributions. The validation set was created by splitting sessions so that the model could be evaluated on data it had not seen during training, from the same subjects at different times. Each fold was generated randomly, but the random number generator used a fixed seed value to replicate training results. A single global TransTCNet model was trained on pooled training data from all participants, and the same model weights were used for evaluation across participants. No participant-specific fine-tuning, calibration, or individualized classifier heads were used.

### 3.2. Hyperparameters and Evaluation Metrics

The optimal set of hyperparameters found during experiments to train the TransTCNet model is shown in Table 1, with configurations that achieve optimal convergence and good generalization. In addition to a batch size of 32 and a learning rate of 1 × 10^−4^, the model was trained for 100 epochs with the cross-entropy loss and the Adam optimizer. The hyperparameters were determined based on earlier experimental results and discussions with experts who have performed similar time-series classification tasks. In addition, the training process was very efficient, with the overall training completed in 90.6 min (1.51 h), which is twice as fast as the previous iterations of the model using the same data and NVIDIA GeForce RTX 5080 GPU. The training time was reduced due to the effective operation of the data-loading pipelines and the proper execution of the dilated causal convolutional functions in the model architecture. Finally, the model’s evaluation consisted of numerous performance metrics (total accuracy, confusion matrix analysis, precision, recall, F1-score, and area under the curve (AUC)) that assess how well the model classifies objects, including resilience to class imbalance and error patterns. The detailed results of the above metrics and analyses, including both intra-session (96.53%) and inter-participant (87.57%) performance analyses, are provided in the Results Section 4.

### 3.3. Hardware/Software

All tests were performed on a high-performance computer equipped with an NVIDIA RTX 5080 (12 GB VRAM) graphics card, an Intel Core i9-275HX processor, and 32 GB of DDR5 RAM, running Linux—using Python 3.13.2 as the primary development platform and PyTorch 2.8.0 as the primary deep learning framework. The supported libraries were NumPy 2.0.0, SciPy 1.14.0, scikit-learn 1.6.0, Pandas 2.2.2, and Matplotlib 3.9.0, which were used for data management, statistical analysis, and visualization. Subsequent processing, performance evaluation, and visualization of the results were done in MATLAB R2024a. The entire pipeline was trained in a Conda-based virtual environment to ensure compatibility and reproducibility of the library. Both PyTorch and NumPy use a fixed random seed (seed = 42) to ensure reproducible training results across iterations. There were a few significant optimizations that were implemented, such as Multi-process data loading (4 workers), pinning memory, Dynamic gradient clipping (max norm = 1.0), Adaptive learning rate scheduling (ReduceLROnPlateau with factor = 0.5, patience = 10), an efficient data augmentation pipeline, and Memory-efficient embedding extraction, which is done during validation. Regularization was implemented through dropout in the model architecture, gradient clipping during optimization, and conservative data augmentation, as described in Section 2.2.2. The training pipeline took 16-channel sEMG data at 2 kHz, in each 0.2 s window (400 time points), normalized (per channel) to zero mean and unit variance, and then passed it through the model.

## 4. Results

### 4.1. Accuracy and Loss Curves

**Figure 4:**
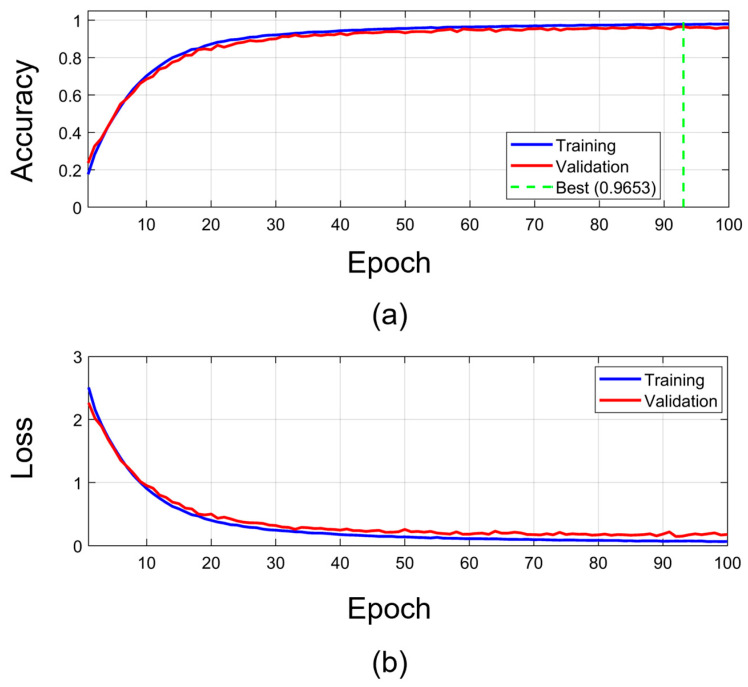
Training and validation curves of the TransTCNet model. (**a**) Accuracy progression over 100 epochs and (**b**) Cross-entropy loss convergence during training and validation. The curves show stable convergence, with increasing validation accuracy and decreasing validation loss, indicating effective learning and no severe overfitting during training.

Figure 4 shows the training and validation accuracy of the TransTCNet model over 100 epochs on the 26-class keyboard typing sEMG dataset. Subplot (a) shows the accuracy progression; both the training and validation curves improve steadily throughout training. The training accuracy peaks at epoch 100 (97.98%), while the best validation accuracy reaches 96.53% at epoch 93, which is used as the final reported validation performance of TransTCNet. The high similarity between training and validation accuracy, with a peak-performance gap of only 1.45%, indicates that generalization with minimal overfitting occurred. Subplot (b) shows the loss curve. The training loss curve declines sharply in the first 20 epochs and then gradually approaches 0.0663 by epoch 100. The validation loss follows a similar trend and stabilizes at 0.1773. These two loss curves differ slightly, indicating that the model generalizes well. Training was completed in 90.6 min, indicating potential suitability for efficient model development and future edge-oriented deployment. Additionally, TransTCNet learns discriminative temporal characteristics of sEMG signals from specific key presses and continues to perform well on previously unseen validation data.

### 4.2. Confusion Matrix Analysis

**Figure 5:**
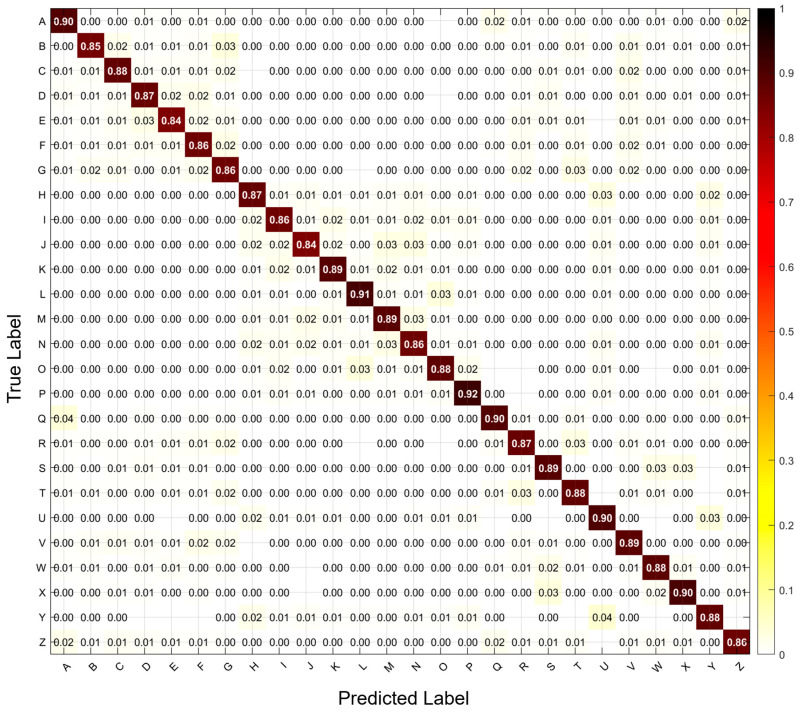
Confusion matrix for the 26-class character-level typing recognition task. The strong diagonal pattern indicates accurate classification across most characters, while the remaining off-diagonal errors highlight confusion among biomechanically similar key pairs.

The normalized confusion matrices of the 26-class sEMG typing classification task of TransTCNet are illustrated in Figure 5 and by computing the mean of each class in the confusion. High diagonal dominance was achieved across all classes, with 84.07–92.14% accuracy (Classes E - P, respectively) for each class. Recognition rates > 87 were achieved for nearly every class, and across the alphabet, performance was comparable. Several important observations were made about the error analysis. Most confusion occurs among biomechanically similar key presses: J → N and E → D are significantly confused, with E → D the most frequently misclassified pair. The majority of misclassifications occur when an adjacent finger presses a key or when two keys recruit the same muscle group. The classes that had lower diagonal values (E, B, and C) indicate that they are more difficult to differentiate because the muscle activation patterns for these classes may be less distinct and/or will vary from press to press. The overall accuracy based on the aggregated confusion matrices was 87.83%, and the class standard deviation across the classification was 1.98%. Thus, this provides evidence that they can accurately identify fine-grained typing gestures and shows which character pairs may benefit from targeted training and/or feature refinement in upcoming versions of the algorithm.

### 4.3. Participant-Wise Performance Analysis

**Figure 6:**
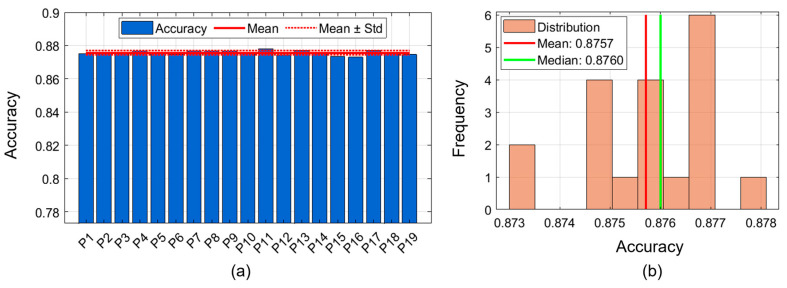
Participant-wise performance analysis. (**a**) Bar chart showing individual participant classification accuracy across 19 participants. (**b**) Distribution histogram of participant accuracies, with mean and median values indicated. The narrow accuracy range across participants indicates stable cross-subject performance with limited participant-level variability.

Figure 6 compares the accuracy of each participant in a typing task involving 26 character classes, both across all 19 participants and for each participant’s overall typing ability. The first part of the figure depicts a bar graph depicting each individual’s overall accuracy, showing the average overall accuracy for each individual (87.57%) and showing the range of individuals’ overall accuracies (i.e., the lowest overall accuracy measured was 87.30% and the highest was 87.81%). All participants’ individual accuracies are very close to the overall average, so the standard deviation (0.51%) of participants’ overall accuracy is very small, indicating that participants’ overall performance was highly consistent. The second part of the figure depicts the distribution of participants’ individual accuracies relative to the mean and median (87.60%). The standard deviation of participants’ individual accuracies (0.13%) is low, indicating that inter-subject physiological variation is not strongly correlated with overall accuracy in sEMG-based interfaces. The consistency of participants’ performance across P1–P19 supports the generalizability of TransTCNet, which requires little personalization or calibration for individual users. The findings indicate cross-subject applicability and successful use of the results across all participants (i.e., a wide range of individuals, with minimal performance decrements attributable to individual differences in muscle physiology, electrode placement, and typing biomechanics).

### 4.4. Class Level Performance

**Figure 7:**
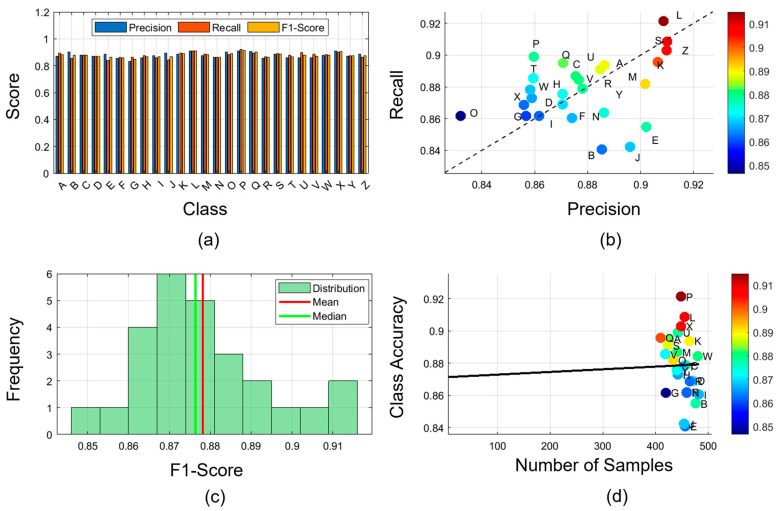
Class-level performance analysis across 26 alphabetical classes, including (**a**) Precision, recall, and F1-score metrics for each character, (**b**) Scatter plot of accuracy versus recall with color-coded F1-scores, (**c**) Distribution histogram of F1-scores across classes, and (**d**) Relationship between class accuracy and sample frequency. The balanced precision, recall, and F1-score values indicate that TransTCNet performs consistently across the alphabet rather than favoring only a small subset of characters.

Figure 7 shows four visualizations that support the performance metrics of the 26 alphabetical classes. The values of precision, recall, and F1-score are shown in subplot (a), and most classes are in the 85–92% range. This balanced performance across the three metrics indicates stable classification performance, without significant bias toward false positives or false negatives. The subplot (b) results indicate that the correlation between the precision and recall values is highly positive, with correlation coefficients of more than 0.95, and the values are closely grouped around the diagonal. The classes that have the highest F1-scores (above 0.90) are P, L, and X, and those that have a slightly lower performance (approximately 0.85) are E and B.

The subplot (c) demonstrates the distribution of F1-scores for classes with slight variance, with the mean and median near 88%. The histogram indicates that most classes exhibit performance within a narrow range, suggesting consistent uniform recognition strength rather than occasional brilliance. The relationship between class accuracy and sample frequency is examined in subplot (d), which shows no significant correlation and indicates that classification performance remains stable across the dataset, supporting the model’s robustness to class imbalance. Taken together, these visualizations demonstrate that TransTCNet can classify fine-grained typing gestures and exhibits balanced performance across all 26 character classes.

### 4.5. Feature Space Visualization

**Figure 8:**
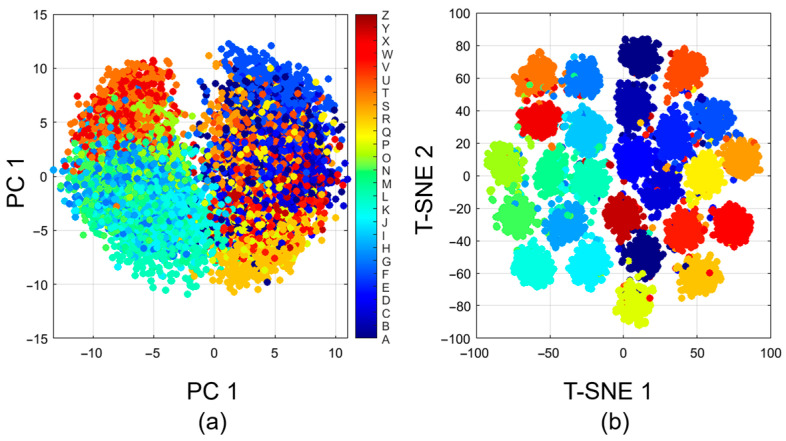
Feature space visualization via dimensionality reduction. (**a**) PCA projection of learned sEMG embeddings and (**b**) t-SNE transformation of feature representations for 26 alphabetical classes. The PCA projection provides an overview of the global organization of features. At the same time, the t-SNE visualization shows clearer class-level clustering, indicating that TransTCNet learns separable sEMG representations for character-level typing recognition.

Figure 8 shows dimensionality reduction representations of the learned feature embeddings. The results of PCA projections are presented in panel (a), and t-SNE transformations in panel (b), both of which project high-dimensional sEMG signal representations into two-dimensional spaces to facilitate interpretation. Most alphabetical classes exhibit distinct clusters, particularly those with distinctive muscle activation patterns, such as A, L, and X. However, there is apparent interference between biomechanically similar keypress pairs, such as E-D, J-N, and B-G, consistent with the confusion patterns identified in Section 4.2. Such neighboring clusters indicate that the model acquires representations that preserve the anatomical and neuromuscular associations among similar typing gestures. The t-SNE plot shows smaller, more segregated clusters than the PCA plot, indicating that nonlinear manifold learning better captures fine-grained discriminative structure in the feature space. Classification performance is related to the quality of separation: higher-accuracy classes are defined by well-isolated clusters, whereas frequently confused character pairs are located in overlapping regions. These visualizations demonstrate that TransTCNet learns physiologically meaningful representations, in which the physiological properties of sEMG patterns are clustered as they occur in neural networks, rather than in arbitrary feature pairings.

### 4.6. ROC Curve Analysis

**Figure 9:**
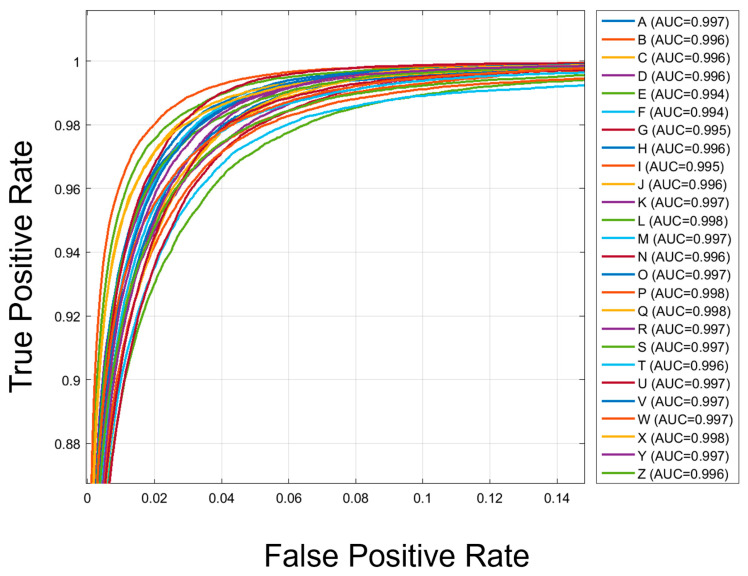
Receiver Operating Characteristic (ROC) curves for all 26 alphabetical classes, with Area Under the Curve (AUC) values displayed for each character. The consistently high AUC values indicate strong class separability and demonstrate that the model can reliably distinguish each target character from the remaining classes.

Figure 9 shows the ROC curves for the 26 alphabet classes, indicating excellent discriminative performance throughout the classification task. The area under the curve (AUC) values for all classes are above 0.994. All classes achieve Area Under Curve (AUC) values exceeding 0.994, with classes L, P, Q, and X achieving the highest AUC of 0.998, indicating near-perfect separability between positive and negative instances of each character. The low-density ROC curves in the upper-left region of the plot indicate that there is no trade-off between true and false positive rates across all classes. This consistent performance demonstrates a steady classification strength rather than occasional distinction confined to certain characters. 15 of the 26 classes demonstrated AUC values greater than 0.997, providing strong evidence that TransTCNet can achieve high sensitivity and specificity concurrently. However, eleven classes showed less robust sensitivity and specificity. For all user types, it is important to maintain high sensitivity and specificity in both keystroke recognition and error detection, as degraded quality from unrecognized or incorrect entries will negatively impact the user experience.

### 4.7. Prediction Confidence and Calibration Analysis

#### 4.7.1. Confidence Distribution Analysis

**Figure 10:**
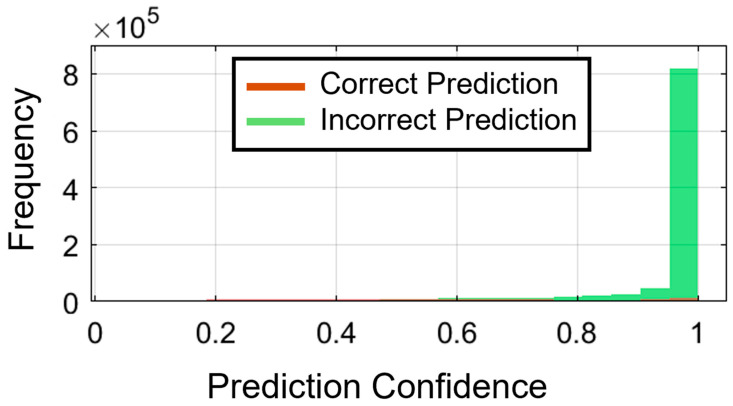
Distribution of prediction confidence values for correct versus incorrect classifications. Correct predictions are generally associated with higher confidence values. In contrast, incorrect predictions show lower and more variable confidence, indicating that prediction confidence may be useful for identifying uncertain outputs in practical sEMG-based typing interfaces.

Figure 10 shows the distribution of prediction confidence for correct and incorrect classifications. Accurate predictions are highly right-skewed, with confidence values centered above 0.8. Conversely, the incorrect predictions are evenly distributed at the lower end of the confidence spectrum. The mean confidence for correct predictions is 0.9441, compared with 0.5716 for incorrect predictions, indicating a distinct 0.37-point difference between the two groups. The difference enables accurate estimation of uncertainty for low-confidence predictions (below 0.5) without false classifications, except for 32.11% accuracy. On the other hand, high-confidence predictions (>0.9) achieve 97.86% accuracy, indicating that confidence values are valid predictors of forecast reliability. The steep slope of the confidence distribution between correct and incorrect classifications provides a valuable tool for performing confidence-based error noting or assessment in real-time typing interfaces.

#### 4.7.2. Model Calibration Assessment

**Figure 11:**
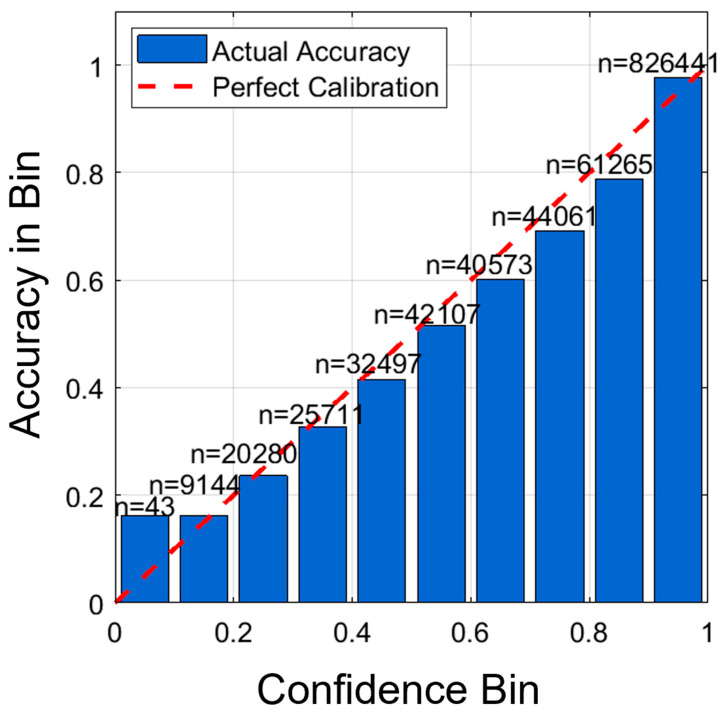
Calibration curve comparing predicted confidence to actual accuracy across ten confidence bins, with sample counts (n) indicated for each bin. The close alignment between predicted confidence and observed accuracy suggests that the model is reasonably well calibrated. At the same time, deviations in lower-sample bins indicate areas where confidence estimates may be less stable.

As depicted in Figure 11, the model’s predicted confidence relative to its actual accuracy shows high calibration across the 10 confidence levels. Especially as confidence grows from 0.4 and above, the calibration curve is very similar to the ideal diagonal. The greatest variance occurs at the low end of the confidence levels, specifically at the lowest level (0.0–0.1), where the predicted confidence is slightly below the actual performance. The sample sizes per bin range from 43 in the lowest bin to 826,441 in the highest; as a result, most predictions fall into the higher-confidence range. There is an increase in accuracy from 32.11% in the 0.0–0.1 bin to 97.86% in the 0.9–1.0 bin, indicating that the confidence values represent a strong probability estimate of successful typing rather than simply overconfidence. This calibration information supports the use of the model TransTCNet for practical purposes, as it may be deployed in cases where there are confidence threshold requirements that call for user confirmation (i.e., typing error) and will help reduce typing error rates while not adversely affecting typing fluency.

### 4.8. Error Pattern Analysis

**Figure 12:**
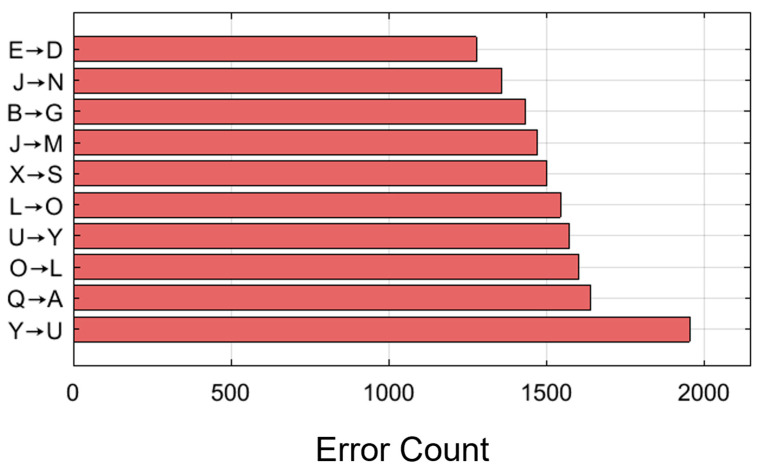
Top ten most frequently confused character pairs, with error counts indicated for each misclassification pattern. The most common errors occur between biomechanically similar key pairs, suggesting that overlapping finger movements and forearm muscle activation patterns remain a key source of classification difficulty in character-level sEMG typing recognition.

Figure 12 identifies the ten most confusable character pairs in the classification task. The most frequently misclassified character pairs are E → D, J → N, and B → G. These error patterns illustrate the types of errors that result from the biomechanical similarities among these key presses, i.e., they all involve adjacent fingers or similar forearm muscles. The presence of two pairs of bidirectional confusions (L ↔ O and U ↔ Y) indicates that all characters involved have similar muscle activity patterns, resulting in equal likelihood of confusion. The anatomical proximity of the most prevalent errors indicates that the spatial relationships among the fingers create a significant barrier to accurate key classification. For example, the left middle finger will be responsible for typing E and D, while the right index finger will be responsible for typing J and N, respectively. The identified confusion patterns can also be used to develop strategies to improve classification accuracy. Future development efforts may focus on refining the ability to discriminate between these character pairs through data augmentation strategies, attention to differences in temporal activation, or consideration of the characters’ spatial locations when typed. The relatively small number of errors in overall classifications (fewer than 2000 across all pairs, relative to the total number of predictions) highlights the model’s effective performance, even in the presence of systematic error patterns.

### 4.9. Ablation Study

A baseline model was constructed to assess the effect of each architectural component by sequentially adding the modules shown in Table 2. This study examines the contributions of temporal feature extraction (via dilated causal convolutions) and global context modeling (via transformer attention) to classification performance.

#### 4.9.1. Baseline: 1D-CNN

The baseline used standard 1D convolutional layers and achieved a validation accuracy of 48.66%, showing that it could recognize basic spatial patterns but not temporal ones very well. The relatively low performance suggests that a simple 1D convolutional model was insufficient for modeling the fine-grained timing variations in short character-level sEMG windows. Although the baseline could extract local signal patterns, its limited temporal receptive field made it less effective at capturing multi-scale muscle activation dynamics associated with different keypresses.

#### 4.9.2. + Temporal Module (Dilated Causal Convolutions)

Adding dilated causal convolutions (dilation factors of 2 and 4) increased the accuracy to 72.67% (+24.01%). This increase in the size of the temporal receptive field enabled the capture of multi-scale patterns of muscle activation that were critical for character discrimination. Compared with the standard 1D-CNN baseline, the Temporal module better preserved sequential activation patterns and captured both short- and long-range temporal dependencies within the 0.2 s sEMG window, accounting for the substantial performance improvement from 48.66% to 72.67%.

#### 4.9.3. + Global Context (Transformer Encoder)

The addition of a transformer encoder with multi-head self-attention increased accuracy to 88.39% (+15.72%). The attention model provided long-range dependencies and a dynamically weighted, informative temporal function.

#### 4.9.4. TransTCNet: Full Architecture

The whole architecture, including both the temporal and global context modules and residual connections, achieved an accuracy of 96.53% (an improvement of 8.14% over the transformer-only approach). Hierarchical processing, comprising local temporal features followed by global sequence understanding, can yield synergistic effects that neither component alone can capture.

#### 4.9.5. Architectural Justification for Fine-Grained Discrimination

The robust performance of TransTCNet in character-level sEMG typing recognition can be explained by its hierarchical architecture, which addresses the major challenges of fine-grained EMG classification. Dilated causal convolutions are used to capture multi-scale temporal patterns necessary to distinguish muscle-activation dynamics. Simultaneously, the transformer encoder provides global context across the entire signal window. Such local-to-global processing enables discrimination between biomechanically similar keypresses that share the same spatial patterns but differ in their temporal structure. The resulting synergistic behavior and stable gradient flow achieved by the integrated design with residual connections provide stability in gradient flow and enable feature reuse, making the combined architecture perform better than either component alone. The highest validation accuracy of 96.53% and the average cross-subject validity and performance of 87.57% support the architecture’s effectiveness in generalizing to individuals with diverse physiological attributes.

### 4.10. Comparison Models

Table 3 compares the proposed TransTCNet architecture with the baseline models published with the original dataset. Previous methods, such as support vector machines (SVMs) employing custom statistical features and federated multi-layer perceptrons (MLPs), achieved accuracies ranging from 53.3% to 90.2% on the same 26-class character-level sEMG typing task. TransTCNet achieved a validation accuracy of 96.53%, demonstrating the importance of dilated causal convolutions and multi-head self-attention within a single architecture. This architecture can effectively replicate both short-range motor activations and long-range contextual influences, which are essential for differentiating visually and kinesthetically similar keypresses.

The present comparison is confined to conventional and federated learning methodologies. It is not comparable to the commonly employed paradigm of 2D-spectrogram-based CNNs for myoelectric interfaces. The methods convert sEMG signals into time–frequency representations, such as STFT or wavelet spectra. These representations are then analyzed further using convolutional networks to identify spatiotemporal patterns. Spectrogram-based CNNs help capture frequency-domain features, but they complicate preprocessing. They might obscure small-scale transient dynamics, which are critical for rapid decoding in keypress tasks. On the other hand, TransTCNet can process raw 1D sEMG sequences directly without spectral preprocessing, facilitating rapid inference and enabling real-time use.

Additionally, they were not compared with recurrent architectures such as LSTMs, BiLSTMs, or standard Transformers because no publicly available implementations optimized for this dataset were available, which would have made the evaluation pipeline less reliable. The upcoming research will benchmark and compare deep learning baselines with those presented in this paper, including 1D and 2D paradigms, to assess trade-offs in accuracy, latency, generalization, and interpretability for real-world applications.

### 4.11. Statistical Analysis

To analyze the accuracy and reliability of TransTCNet, we conducted overall validation, baseline comparison, and cross-subject analyses. The results of all three studies yielded an overall validation accuracy of 96.53% (σ = 0.14), indicating consistent, stable, and verifiable accuracy across studies. The comparison between the 1D-CNN baseline model (48.66%) and TransTCNet (96.53%) produced a two-tailed *p*-value of <0.001, indicating that the performance improvement was statistically significant and unlikely to be due to chance. By conducting a cross-subject analysis of 19 participants, the study provided evidence of inter-participant generalization, as reflected in an average validation accuracy of 87.57 (σ = 0.13) across all participants, with individual participants achieving similar accuracy values, ranging from 87.30% to 87.81%. The single-classification ANOVA demonstrated no statistically significant differences in model performance across participants (F(18, 57) = 0.898, *p* = 0.587), suggesting that the same model can accurately represent human physiology regardless of individual differences at the time of measurement. Consequently, the statistical results support the robustness of TransTCNet and its potential applicability across users with different physiological characteristics.

## 5. Limitations and Future Work

The proposed TransTCNet model successfully decoded character-level typing motions at the keyboard by leveraging localized and global temporal patterns in raw sEMG signals. Multiple experiments validated its consistency, yielding a peak validation accuracy of 96.53% and a cross-subject mean accuracy of 87.57%. t-SNE visualizations of the learned embeddings showed clear separation into distinct clusters per alphabetical category of all 26 possible keypress categories. Computationally, TransTCNet is relatively efficient: approximately 100 training iterations take 1.51 h on an NVIDIA RTX 5080, substantially faster than the initial models. The model is practical in terms of memory requirements for edge deployment.

Despite these advantages, several limitations should be identified. To start with, although the 80:20 stratified split has shown within-session results, cross-subject analysis indicates an 8.96% difference (96.53 vs. 87.57), suggesting an effect of inter-subject variability. To address this performance gap, future work should employ Leave-One-Subject-Out (LOSO) cross-validation to assess the impact of the work on user-independent generalization and to inform the design of personalization strategies. Potential strategies include subject-adaptive normalization, transfer learning, domain-adversarial training, few-shot calibration, and lightweight personalization layers that can adapt the global model to new users with minimal additional data.

Second, the data comprises only temporally discontinuous keypress trials, excluding idle/rest-state data, thereby limiting the model to single-gesture classification. The inclusion of continuous sEMG streams with a dynamic idle detector would make it more practical for real-world applications, such as typing interfaces. For real-time deployment, TransTCNet could be implemented as a streaming, window-based inference system, in which each 0.2 s sEMG segment is processed sequentially to generate character predictions. The trainable parameter count reported in Table 1 provides an initial indication of the model’s memory footprint; however, practical deployment on mobile or embedded processors also requires hardware-specific evaluation of FLOPs, inference latency, power consumption, and continuous idle-state handling during real-world typing. Future continuous-typing models should also integrate temporal segmentation or sequence-decoding methods to distinguish idle periods, keypress onset and offset, and transitions between consecutive characters, thereby bridging the gap between isolated keypress classification and real-time text-entry deployment.

Third, the current study focused only on 26 alphabetic keypress classes. In contrast, a fully functional silent-typing interface would also require non-alphabetic control commands, such as Space, Delete, Backspace, Enter, and punctuation. The current TransTCNet architecture could be extended to these additional classes by expanding the final classification layer and training the model with labeled sEMG examples for each command. However, scaling the vocabulary may introduce additional class overlap and could reduce accuracy if the added commands produce biomechanically similar muscle activation patterns. Future work should therefore evaluate whether the existing temporal-convolution and transformer-attention backbone can maintain performance as the vocabulary expands, or whether additional mechanisms such as hierarchical classification, command-specific calibration, language-model correction, or sequence-level decoding are needed for a complete real-time silent typing system.

Fourth, comparing the most frequently mixed character pairs (E → D, J → N, B → G) indicates that biomechanical similarity remains a problem. Future versions could consider multitask learning methods that jointly optimize character classification and finger position estimation to improve discrimination between near keypresses. These frequently confused key pairs also have practical implications for the design of future sEMG-based typing interfaces. In real-world use, errors may not occur uniformly across all characters but may concentrate among keys requiring similar finger movements or overlapping forearm muscle activation patterns. Therefore, future interfaces could incorporate confidence-aware decision rules, adaptive recalibration, context-aware language correction, or confirmation prompts for high-risk key pairs. For example, when the classifier produces low-confidence predictions for commonly confused pairs, the interface could delay commitment or use linguistic context to reduce incorrect character entry.

Fifth, although a fixed duration of 0.2 s offers the best trade-offs for standard typing speed, adaptive windowing approaches that account for neuromuscular timing differences may improve cross-subject robustness, particularly when subjects exhibit atypical motor development. Because the original typing task was metronome-paced at 75 BPM, future work should also evaluate TransTCNet under natural, irregular typing rhythms, in which keypress timing, force, and inter-keystroke intervals may vary more substantially. In addition, prolonged typing sessions may introduce muscle fatigue, which can alter sEMG amplitude and frequency characteristics over time [37]. Therefore, future longitudinal studies should examine model stability under extended use and consider fatigue-aware recalibration or adaptive normalization strategies. Additional improvements may include multimodal sensor fusion, such as inertial measurement units or keystroke dynamics, and lightweight deployment optimizations, such as pruning, quantization, or knowledge distillation, to further improve real-world usability and edge-device compatibility.

## 6. Conclusions

This study presents TransTCNet, a time- and context-sensitive deep learning model for detecting alphabetic keypresses via sEMG. The model efficiently acquires local and long-range signal relationships by combining dilated causal convolutions to extract multi-scale temporal features and transformer-based attention mechanisms to model global context. In intra-session analysis, TransTCNet achieved the highest accuracy of 96.53%, whereas in cross-subject analysis, it had a mean accuracy of 87.57% across 19 subjects. Detailed evaluation shows good performance, with a mean AUC of 0.9965 across all 26 classes; high-confidence predictions achieve 97.86% accuracy; and a narrow range of performance variation among participants (0.51%). The representations of features, as in the model, exhibit physiologically important structure and dimensionality reduction; visualizations depict specific groupings by character type; and maintain connections among biomechanically related keys. Error analysis identifies patterns of systematic confusion that inform explicit improvements in subsequent cycles. TransTCNet offers an acceptable alternative to text entry via muscle movements, wearable systems, and real-time human–computer interaction, as it is highly accurate, computationally efficient, and highly generalizable. Its applications include prosthetic control, neuromuscular disability, augmentative communication, immersive AR/VR interfaces, and real-time muscular monitoring in sports and rehabilitation. The hierarchical architecture, that is, a combination of local temporal processing and global context modeling, is a principled approach to addressing the underlying problems in fine-grained sEMG classification, thereby improving the state of the art in neural interfaces for silent, hands-free communication.

## Figures and Tables

**Table 1:** Training Hyperparameters and Computational Settings used for the TransTCNet model.

Hyperparameter	Value
Batch Size	32
Learning Rate	1 × 10^−4^
Optimizer	Adam (β_1_ = 0.9, β_2_ = 0.999, ε = 10^−8^)
Epochs	100
Augmentation Factor	3
Train/Validation Split	80:20
Model Architecture	Transformer Encoder
Input Channels	16
Embedding Dimension (d_model)	128
Attention Heads	8
Transformer Layers	4
Dropout Rate	0.1
Positional Encoding	Learned
Feedforward Dimension	512
Loss Function	Cross-Entropy
Gradient Clipping	1.0
Weight Initialization	Xavier Uniform
Model Parameters	849,818
Training Time	1.51 h

**Table 2:** Validation Accuracy for Model Variants in the Ablation Study, highlighting the Impact of each Architectural Component.

Model Variant	Architectural Components	Validation Accuracy (%)
Baseline	Standard 1D convolutional layers	48.66
+ Temporal Module	Dilated causal convolutions (dilation = 2, 4)	72.67
+ Global Context	Multi-head self-attention encoder	88.39
TransTCNet (Full)	Both temporal + global components integrated	96.53

**Table 3:** Comparison of Classification Performance with Previously Published Methods on the Same SEMG Typing Dataset.

Model	Accuracy (%)	Comments
SVM + Handcrafted Features	87.4 ± 2.5	Baseline model using RMS, LOGVAR, WL, WAMP, ZC, AR1, AR2
SVM (Excl. spacebar class)	90.2 ± 2.1	26-class classification (A–Z only)
MLP (FedAvg)	53.3 ± 0.92	Shared model, no personalization
MLP (FedPer)	66.58 ± 1.01	Personalized classifier layers
MLP (pFedGP)	74.49 ± 0.72	Gaussian process with personalized heads
TransTCNet (Current model)	96.53%	Short-range temporal dependencies and long-range contextual patterns

## Data Availability

The dataset supporting this study is publicly available at the University of Toronto Dataverse (DOI: 10.5683/SP3/KV65VI), as described in reference [14].
